# The Scarlet Fever

**Published:** 1883-05

**Authors:** 


### THE SCARLET FEVER.

IT IS as unnecessary for a child to die of the scarlet fever as it is that it should be blind with a cataract. Let us see. At any time before the body has finished its effectual struggle we are able to help it, not by wonderful medicines, but but the knowledge of anatomy, and the application of common sense. We consult the sympathetic nerve, and do what it commands us to do. We must give this child salt when it wants it; we must give it acid when it has fever and anxiously craves it—not vinegar, but lemon juice, because the first coagulates albumen, and the other does not, on account of the surplus oxygen which it contains. To imitate the soothing mucus in the intestines, which is now wanting, and to give some respiratory food at the same time, we add some gum arabic. To restore and relieve the injured nerve, we apply moist warmth. In practice we can fulfill all this with the following simple manipuiations: Undress the child and bring it to bed at the very first sign of sickness. Give it, if it has already fever, nothing but sourish warm lemonade with some gum arabic in it. Then cover its abdomen with some dry flannel. Take a well folded bed sheet and put in boiling hot water; wring it out dry by means of dry towels, and put this over the flannel on the child's abdomen. Then cover the whole, and wait. The hot cloths will, perhaps, require repeated heat. According to the severity of the case, and its stage of progress, perspiration will commence in the child from ten minutes to two hours. The child is then saved; it soon falls to sleep. Soon after the child awakes, it shows slight symptoms of returning inclination for food; help its bowels, if necessary, with injections of oil, soap and water, and its recovery will be as steady as the growth of a green house plant, if well treated. Of course, if the child was already dying, nothing could save it, or if it has already effusions in the lining of the heart or brain, it is much better it should die. But if the above is applied in due time, under the eyes and direction of a competent physician, I will guarantee that not one in a hundred children will ever die of scarlet fever. I know this will startle some of my readers, especially those who have lost children already, but I shall go still farther. I maintain that a child will never get the scarlet fever if properly treated. If a child has correctly mixed blood, it will not catch the disorder if put in bed with a sick child.

This is still more startling, but nothing is easier of proof.—*Good Health.*

